# Simultaneous Identification of Clinically Common *Vibrio parahaemolyticus* Serotypes Using Probe Melting Curve Analysis

**DOI:** 10.3389/fcimb.2019.00385

**Published:** 2019-11-14

**Authors:** Minxu Li, Yixiang Jiang, Xiaolu Shi, Yinghui Li, Min Jiang, Yiman Lin, Yaqun Qiu, Le Zuo, Yinhua Deng, Zeren Lin, Yiqun Liao, Qingge Li, Qinghua Hu

**Affiliations:** ^1^College of Life Sciences and Oceanography, Shenzhen University, Shenzhen, China; ^2^Shenzhen Center for Disease Control and Prevention, Shenzhen, China; ^3^College of Life Sciences, Sichuan University, Chengdu, China; ^4^School of Life Sciences, Xiamen University, Xiamen, China

**Keywords:** *Vibrio parahaemolyticus*, vibriosis, molecular serotyping, serogroup, identification, multiplex ligation reaction based on probe melting curve analysis

## Abstract

The dynamic nature of *Vibrio parahaemolyticus* epidemiology has presented a unique challenge for disease intervention strategies. Despite the continued rise of disease incidence and outbreaks of vibriosis, as well as the global emergence of pandemic clones and serovariants with enhanced virulence, there is a paucity of molecular methods for the serotyping of *V. parahaemolyticus* strains to improve disease surveillance and outbreak investigations. We describe the development of a multiplex ligation reaction based on probe melting curve analysis (MLMA) for the simultaneous identification of 11 clinically most common *V. parahaemolyticus* serotypes spanning a 10-year period. Through extensive sequence analyses using 418 genomes, specific primers and probes were designed for a total of 22 antigen gene targets for the O- and K- serogroups. Additionally, the *toxR* gene was incorporated into the assay for the confirmation of *V. parahaemolyticus*. All gene targets were detected by the assay and gave expected Tm values, without any cross reactions between the 11 clinically common serotypes or with 38 other serotypes. The limit of identification for all gene targets ranged from 0.1 to 1 ng/μL. The intra- and inter-assay standard deviations and the coefficients of variation were no more than 1°C and <1% respectively, indicating a highly reproducible assay. A multicenter double-blind clinical study was conducted using the traditional *V. parahaemolyticus* identification workflow and the MLMA assay workflow in parallel. From consecutive diarrheal stool specimens (*n* = 6118) collected over a year at 10 sentinel hospitals, a total of 153 *V. parahaemolyticus* isolates (2.5%) were identified by both workflows. A total agreement (kappa = 1.0) between the serotypes identified by the MLMA assay and conventional serological method was demonstrated. This is the first molecular assay to simultaneously identify multiple clinically important *V. parahaemolyticus* serotypes, which satisfies the acute need for a practical, rapid and robust identification of *V. parahaemolyticus* serotypes to facilitate the timely detection of vibriosis outbreaks and surveillance.

## Introduction

*Vibrio parahaemolyticus* is a halophilic bacterium that thrives in estuarine, marine, and coastal waters and is recognized as the primary cause of gastroenteritis associated with the consumption of seafood and marine products worldwide (Cabrera-Garcia et al., 2004; Mclaughlin et al., 2005; Letchumanan et al., 2014). In China, *V. parahaemolyticus* has been the leading cause of foodborne outbreaks and infectious diarrhea among adults, particularly in coastal cities (Li Y. et al., 2014; Liu et al., 2018). According to the Foodborne Disease Outbreak Surveillance System (FDOSS) in China, a year-on-year increase in the number of outbreaks due to *V. parahaemolyticus* was observed between 2011 and 2016, having caused more outbreaks than other major foodborne pathogens for each given year (Liu et al., 2018). Similarly, a clear upward trend in the incidence of foodborne vibriosis was observed in the United States in recent years (Newton et al., 2012; Marder et al., 2017). Indeed, the incidence of vibriosis has been rising, and there is a growing body of evidence that this could become increasingly common due in part to the rising sea water temperatures from the effects of global warming that could favor its spread (Baker-Austin et al., 2010, 2017; Vezzulli et al., 2013). Currently, 13 O- serogroups and 71 K- serogroups are recognized based on the antigenic properties of the somatic (O) and capsular (K) antigens, respectively (Iguchi et al., 1995; Oliver and Jones, 2015). *V. parahaemolyticus* infections are recognized as a multiserogroup affliction caused by diverse serotypes (Bhuiyan et al., 2002), leading primarily to acute gastroenteritis, and occasionally, wound infection and septicemia (Daniels et al., 2000). In 1996, the emergence of a more virulent serotype O3:K6 was subsequently being recognized as a pandemic clone owing to its rapid dissemination and the cause of numerous gastroenteritis outbreaks globally (Nair et al., 2007). To date, the pandemic clone and its serovariants are known to represent 49 serotypes, which are widely distributed among 22 countries in Asia, Europe, America and Africa (Han et al., 2016). In China, up to 27 pandemic serovariants have been found amongst clinical samples, in which O3:K6, O4:K68, and O1:KUT predominated (Han et al., 2017). Hence, the serotyping of *V. parahaemolyticus* has played a pivotal role in significantly advancing our understanding of its epidemiology.

Currently, the serotyping of *V. parahaemolyticus* is performed routinely using the traditional serum slide agglutination method. However, this conventional serological method suffers from several major practical and technical drawbacks, including tedious and time-consuming procedures, ambiguous results arising from cross reactivity, batch-to-batch inconsistencies, and subjectivity in results interpretation. Despite these drawbacks, the development of molecular methods for the serotyping of *V. parahaemolyticus* has been scarce, with only a few methods described to-date. A conventional PCR-based method has been described for the detection and identification of *V. parahaemolyticus* O-serogroups based on O-serogroup genetic determinants (OGDs) (Chen et al., 2012), which was subsequently extended to include three additional provisional O-serogroups, but without any K- serogroups (Guo et al., 2017). Another conventional PCR was developed to detect an arbitrary marker for the identification of a single serotype O3:K6 (Yeung et al., 2003). A MALDI-ToF mass spectrometry method have also been explored for the serotyping of *V. parahaemolyticus*, albeit with usage limited to differentiate O4:K8 and non-O4:K8 isolates (Li et al., 2018). These methods suffer from practical limitations such as low throughput, cumbersome procedures, ambiguous results arising from gel electrophoresis, and the ability to identify limited serogroups and serotypes.

In recent years, multiplex ligation reaction based on the probe melting curve analysis (MLMA) has been shown to be a robust and high throughput method that has been successfully applied for the simultaneous detection of 10 bacterial pathogens (Jiang et al., 2017). This method combines the use of fluorescence color and melting temperature (Tm) as a virtual two-dimensional label that allows the homogenous detection of multitudes of gene targets (Liao et al., 2013). Based on the principles of MLMA, we present the first molecular assay to simultaneously identify 11 clinically most common *V. parahaemolyticus* serotypes. Coupled with the use of basic laboratory protocols and inexpensive equipment, the current assay offers a practical solution to facilitate the early identification of *V. parahaemolyticus* serotypes, which is an essential strategy for the successful prevention and control of vibriosis incidence and outbreaks.

## Materials and Methods

### Reference Bacterial Isolates

A total of 244 reference bacterial isolates were used for the initial development and evaluation of the MLMA assay. This comprised of reference isolates (*n* = 176) representing 11 clinically most common *V. parahaemolyticus* serotypes during a 10-year period (2007–2017) from the Shenzhen Center for Disease Control and Prevention isolates collection (Table 1); and reference isolates (*n* = 66) representing additional 38 serotypes for detecting cross reactions (Table S1). For completeness, two rare serogroups absent during the 10 year period (O7 and O12) were incorporated into the assay using *Escherichia coli* TOP10 strains (*n* = 2) containing serogroup-specific genes (O7-*wvcN*, O12-*wvcP*) cloned into a pUC57 vector (Sangon Biotech Co. Ltd., Shanghai, China).

**Table 1:** Reference isolates (*n* = 176) representing 11 clinically most common *V. parahaemolyticus* serotypes over a 10-year period (2007–2017).

**Serotype**	**Number of isolates**
O3:K6	20
O4:K8	20
O3:K29	20
O1:KUT	3
O1:K56	20
O1:K36	20
O4:K9	20
O4:K68	20
O1:K25	20
O2:K3	10
O3:KUT	3

### Bacterial Culture and Conventional Serotyping

Stool specimens from sentinel hospitals in the multicenter double-blind study were enriched in alkaline peptone water [(pH 8.6); 3% NaCl] and incubated at 37°C for 16 h on a shaker; then streaked onto *Vibrio* chromogenic agar incubated at 37°C for 12 h for single colonies (Guangdong Huankai Microbial Science and Technology, Guangzhou, China).

Suspected colonies were picked and streaked onto triple sugar iron slants (Guangdong Huankai Microbial Science and Technology, Guangzhou, China) and incubated at 37°C for 16 h followed by serotyping by serum slide agglutination tests using commercial antisera (Denka Seiken, Tokyo, Japan) according to the manufacturer's protocol and the Chinese National Food Safety Standards: Food Microbiological Examination *Vibrio parahaemolyticus* Testing, GB 4789.7-2013. Two *E. coli* TOP10 strains with pUC57 vector for O7 and O12 serogroup-specific genes were cultured onto LB medium (Guangdong Huankai Microbial Science and Technology, Guangzhou, China) and incubated at 37°C on a shaker for 16 h.

### DNA Extraction

DNA templates for reference and clinical isolates were prepared using the boiled lysates method. A single colony was suspended in 50 μl of distilled water, then boiled at 100°C for 8 min and centrifuged at 12,000×*g* for 10 min, with supernatant used as DNA template. Plasmid DNA templates were extracted from two *E. coli* TOP10 strains using the SanPrep Plasmid MiniPrep Kit (Sangon Biotech Co. Ltd., Shanghai, China). The concentration of all DNA templates was measured spectrophotometrically (Nanodrop ND-1000, Wilmington, Delaware, USA).

### Primers and Probes Design

In the current assay, the target gene loci for of O- and K- antigens of *V. parahaemolyticus* were located between *dgkA* and *gmhD* genes (Chen et al., 2012) and between *gmhD* and *rjg* genes (Chen et al., 2010), respectively, as previously determined. We sequenced 418 genomes and performed sequence analyses to identify polymorphic sites specific among the serogroup antigen genes using multiple alignments (MEGA7.0). The *toxR* gene was incorporated into the assay as a target gene for the confirmation of *V. parahaemolyticus* as previously described (Jiang et al., 2017). Designed primers (Primer Premier v5.0) and probes (DNA folding software, http://unafold.rna.albany.edu/?q=mfold/DNA-Folding-Form) were evaluated using the BlastN algorithm (https://blast.ncbi.nlm.nih.gov/Blast.cgi). Fluorogenic probes were labeled at 5′ end with reporter fluorophores carboxy fluorescein (FAM), carboxy-X-rhodamine (ROX), or indodicarbocyanine (Cy5) and with Black Hole Quencher sequestering molecule at the 3′ end. All primers and probes were synthesized by Sangon Biotech Co., Ltd. (Table 2).

**Table 2:** MLMA assay for the identification of *V. parahaemolyticus* O- and K- serogroups in a two-tube system.

**Serogroup**	**Gene loci**	**Tube**	**Fluorescence channel (Tm/^**°**^C)**	**Sequence** **(5^**′**^-3^**′**^)**	**Hybridization-ligation oligonucleotide probe sequence (5^**′**^ → 3^′^)^a^**
O1	*wvaG*	1	(ROX,59.5)	L	**GTGGCAGGGCGCTACGAACAAT**CCTAACGACACTGGCTGCTGGTCCGTGACGACAACATAACCACGTCTGAACCAGAACTGATACT
				R	GTGTCATCAAGATACAGTATCAAAGATCATGAG**TGAGATTGGATCTTGCTGGGC**
O2	*wvaR*	1	(ROX,70.0)	L	**GTGGCAGGGCGCTACGAACAAT**CCTAACGACTCTAGCTGCTCGTTCGTGACGCACCCAAGAATGGCAACATGTTATTACCATAAGTC
				R	GAGACAACCGCAGTATTACAGTCCC**TGAGATTGGATCTTGCTGGGC**
O3/O13	*VP0208*	1	(ROX,66.0)	L	**GTGGCAGGGCGCTACGAACAAT**CCTAACGACTCTGTCTTCTCGTTCGTGACGATGTAAGGAAACGGATTTATGTATAGTGTCACTTGA
				R	CGAGGATAATTATACTAAAAGTTTGCATTTAGGTGTTC**TGAGATTGGATCTTGCTGGGC**
O4	*orf16*	1	(ROX,54.0)	L	**GTGGCAGGGCGCTACGAACAAT**CCTAACGACTATGGCTTCTCGTTGGTGACGCCTGTGCATTACTTTTTTACAGCCTTTACGTTATTTCA
				R	GATCAGCCTCATCTGGTATTTACAATTCCATT**TGAGATTGGATCTTGCTGGGC**
O5	*wvcA*	1	(ROX,63.5)	L	**GTGGCAGGGCGCTACGAACAAT**CCTAACGACTCTAGCTGCTTGTTCGTGACGCCTCAAGAATCTTATGTGTTAGCAGTGTCATGG
				R	CGCTGGATGATTAGAGATGTCGAAGA**TGAGATTGGATCTTGCTGGGC**
O6	*wvcJ*	1	(FAM,65.0)	L	**GTGGCAGGGCGCTACGAACAAT**CCTATCGGTCCTTTATCGCTCACCCTTCACCGGCATCTCAATGTTAAACGGTATGTTTGCAATCA
				R	GTATTTACGATAAGCGCATAGACACTATGTTTC**TGAGATTGGATCTTGCTGGGC**
O7	*wvcN*	1	(FAM,57.0)	L	**GTGGCAGGGCGCTACGAACAAT**CCTATCCGTTCTTTATCGCTCAGCCTTCATCGGCATTGAACCAGACCGTGTGGCCAAGCCAATTATTG
				R	ATCTTGGATGACTGTTCACCTGATGA**TGAGATTGGATCTTGCTGGGC**
O8	*wvdG*	1	(FAM,70.0)	L	**GTGGCAGGGCGCTACGAACAAT**CCTATCGGTCCTTCATCGCTCGGCCTTCACCGGGCCCAACGAAGCAGAGTCAGCATACAAGTTA
				R	GGTATTATTCCGCATTACAACAATAAGAACGAC**TGAGATTGGATCTTGCTGGGC**
O9	*wvaH*	1	(FAM,61.0)	L	**GTGGCAGGGCGCTACGAACAAT**CCTATCGGTCCTTCATGGCTCAGTCTTCACCGGGCTATCTCAAGATGAATCTGAGTGTATTGCAAAAG
				R	TTTATCGATTGATTATAACGAGCGGAACGGAT**TGAGATTGGATCTTGCTGGGC**
O10	*wvcP*	1	(FAM,74.5)	L	**GTGGCAGGGCGCTACGAACAAT**CCTATCGGTCCTTCATCGCTCAGCCTTCACCGGCGCGAGTCTCTCTGAAGGATATGATTCATTAATG
				R	ACGGTAAAAGAGCTGCGCGGTTTTAT**TGAGATTGGATCTTGCTGGGC**
O10	*orf9*	1	(ROX,75.0)	L	**GTGGCAGGGCGCTACGAACAAT**CCTAACGACTCTGGCTGCTCGTTCGTGACGTGTATCTGAACGATTAATTCCCAATGGCT
				R	TGCCGTCTGAGTCGATTTACCACTCCAT**TGAGATTGGATCTTGCTGGGC**
O11	*wvdB*	1	(ROX,57.0)	L	**GTGGCAGGGCGCTACGAACAAT**CCTAACGACTCTAGCTTCTCGTTAGTGACGGATGTATCTCTAGGTTGTTCGGGATACGTATATGG
				R	TCTTTGGCTTGCTCACATGATGATAGAG**TGAGATTGGATCTTGCTGGGC**
O12	*wvcP*	1	(FAM,57.0)	L	**GTGGCAGGGCGCTACGAACAAT**CCTATCGCTCCTTCATAGCTCAGACTTCATCGGACCGTCATTAATGAATCATAGCCTTCCATGAGT
				R	GCTTGATTATACTGTTTGATAATAGCGCTATAACCT**TGAGATTGGATCTTGCTGGGC**
VP	*toxR*	1	(CY5,60)	L	**GTGGCAGGGCGCTACGAACAAT**CCTACGGTGAGGACCTTTGCAGATTGGCATCACCCAACCAGAAGCGCCAGTAGTACCTGAAAAAGCA
				R	CCTGTGGCTTCTGCTGTGAATCCTTGGATT**TGAGATTGGATCTTGCTGGGC**
IC	*SUC2*	2	(ROX,50.5)	L	**GTGGCAGGGCGCTACGAACAAT**CCTAACGACTCTATCTGCTTGTTAGTGACGGATCGCATGACTCAGTCATCGTGAAA
				R	GAAAGGCACAACTTTGTAGAGATTTCTGT**TGAGATTGGATCTTGCTGGGC**
K3	*VP24500037*	2	(FAM,75.0)	L	**GTGGCAGGGCGCTACGAACAAT**CCTATCGGTCCTTCATCGCTCAGCCTTCACCGGTTCTTGCTGACGATCTATGTGTTAACGTAG
				R	ATGGTGATGGCGTTCTTCAGCAGATGGTGA**TGAGATTGGATCTTGCTGGGC**
K6	*VP0223*	2	(ROX,74.5)	L	**GTGGCAGGGCGCTACGAACAAT**CCTAACGACTCTGGCTGCTCGTTCGTGACGCCGTTAGAACCTAAGTCTAATTATGCAGTCA
				R	CTGGGCTATATTTCTATGACAGTCGCGTAATAG**TGAGATTGGATCTTGCTGGGC**
K8	*VPBB0234*	2	(ROX,70.0)	L	**GTGGCAGGGCGCTACGAACAAT**CCTAACGACTCTAGCTGCTCGTTCGTGACGGAACTTGATTGAAGCAAGGGAACATTCTTT
				R	CGGTGAGTATGATTTAATACATTGTCACTTC**TGAGATTGGATCTTGCTGGGC**
K9	*VP13500017*	2	(FAM,66.5)	L	**GTGGCAGGGCGCTACGAACAAT**CCTATCGGTCCTTTATCGCTCACCCTTCACCGGCGGAGTGATTATAAGGAGGAGTGCTATAATG
				R	TGGGTTCGGGAATCGGTGTCAGTGTTAA**TGAGATTGGATCTTGCTGGGC**
K25	*VP13200012*	2	(ROX,67.0)	L	**GTGGCAGGGCGCTACGAACAAT**CCTAACGACTCTGTCTTCTCGTTCGTGACGGCTTATCTAGTCGTTCTTCATTTGGTGAGAAAG
				R	CTTTCAACTCCAAAAGTATCGTGATTAGAA**TGAGATTGGATCTTGCTGGGC**
K29	*VP24700016*	2	(ROX,64.0)	L	**GTGGCAGGGCGCTACGAACAAT**CCTAACGACTCTAGCTGCTTGTTCGTGACGTGATAAGTATTCTTTGATATCGAAAGTGGCGA
				R	GTGTTTACAATAAGAAGATTAAAATTGAGAG**TGAGATTGGATCTTGCTGGGC**
K36	*VP10400014*	2	(FAM,71.0)	L	**GTGGCAGGGCGCTACGAACAAT**CCTATCGGTCCTTCATCGCTCGGCCTTCACCGGTTGAAACAAACTATAGCTTCAGAGTTTCCA
				R	ATTAGTTTCAAAAATATTCTTGAAAGTAAG**TGAGATTGGATCTTGCTGGGC**
K56	*VP33400015*	2	(ROX,60.5)	L	**GTGGCAGGGCGCTACGAACAAT**CCTAACGACACTGGCTGCTGGTCCGTGACGTAGAACACTCAAACCGGAAGTTCATCGCAA
				R	GAATGGATGCTGATGATATTTCAGAGCCA**TGAGATTGGATCTTGCTGGGC**
K68	*VP16100014*	2	(ROX,54.5)	L	**GTGGCAGGGCGCTACGAACAAT**CCTAACGACTATGGCTTCTCGTTGGTGACGCGATACTAATGACTCAGATGTATGCCCAGGATT
				R	TTTACAGAAATGTGGGGCCAAGAAAGTTA**TGAGATTGGATCTTGCTGGGC**

a *Bold typeface indicates universal primer sequences used during the LATE-PCR amplification step (L, Left ligation oligonucleotide, limiting primer; R, Right ligation oligonucleotide, excess primer); whereas fluorescent detection probe sequences are underlined*.

### Development of the MLMA Assay

The MLMA assay involves two main steps as previously described (Jiang et al., 2017): (1) a hybridization-ligation process; and (2) a PCR amplification and melt curve analysis. During (1) hybridization-ligation, a pair of oligonucleotide probes hybridizes with the flanking sequences on either side of the target gene loci, which is ligated only upon complete hybridization. (2) Using a universal PCR primer pair via Linear After-The-Exponential (LATE) PCR, the ligated product is first amplified then hybridized with fluorescently labeled detection probes. This allows *V. parahaemolyticus* O- and K- serogroups to be determined based on unique melting temperatures (Tm) obtained using a melt curve analysis established by corresponding fluorescent detection probes in the respective fluorophore channels.

The hybridization and ligation reaction was performed using a T3 Thermocycler (Biometra, Germany). Each 10 μl reaction mix contained a 1.5 μl of ligation probe mix (2.5–4 fmol of each hybridization oligonucleotide probe, Table 2), 1U Taq DNA ligase and 1 μl DNA ligase buffer (New England Biolabs, Beijing, China), 1.5 μl sterilized water, and 5 μl DNA template. Reaction conditions include an initial DNA denaturation step at 95°C for 5 min containing only genomic DNA and ligation probe mix and allowed to reach 75°C before the addition of the remainder of reaction mixture (3.5 μl). This is followed by an incubation step at 60°C for 80 min and 95°C for 5 min. The PCR amplification and melting curve analysis was performed on a Bio-Rad CFX 96 real-time PCR system (Bio-Rad Inc., Hercules, CA). Each 50 μl reaction mixture consisted of 1× PCR buffer, 3 μl MgCl_2_ (25 mM), 4 μl of deoxynucleoside triphosphate (2.5 mM), 1U of Taq polymerase (TaKaRa Biotech Co., Dalian, China), 0.3μl limiting primer (2.5 μM), 0.3μl excess primer (50 μM), 0.20 μM fluorogenic probes (Table 2), and 5 μl of ligation product from the hybridization-ligation step. Amplification began with a denaturation step at 95°C for 3 min, followed by 38 cycles of 95°C for 5 s, 56°C for 15 s, and then 74°C for 15 s. This is followed by melt curve analysis that began with a denaturation step at 95°C for 2 min, hybridization at 40°C for 2 min, and a stepwise temperature rise from 40°C to 85°C at 1°C per step with 5 s for each step. Fluorescence from the FAM, ROX, and Cy5 channels were obtained and recorded automatically during the 38 amplification cycles, where a melt curve analysis was processed through the CFX Manager 3.0 software.

### Analytical Performance of the MLMA Assay

The limit of identification was determined using a series of 10-fold dilutions in triplicates (from 10 to 0.01 ng/μl) of purified DNA templates from isolates corresponding to *V. parahaemolyticus* O- and K- serogroups. The reproducibility of the assay was evaluated using two sets of 10-fold serial dilutions (100–10 ng/μl) of DNA templates measured in triplicates. The standard deviations and coefficient of variation values of the intra-assay and inter-assay were calculated.

### Multicenter Double-Blind Clinical Study

A multicenter double-blind clinical study was conducted throughout 2018 from 10 sentinel hospitals in Shenzhen to evaluate the MLMA assay compared to the conventional serotyping method. Suspected isolates from diarrheal stool samples were analyzed using the traditional *V. parahaemolyticus* identification workflow and the MLMA assay workflow in parallel and double-blinded. In the traditional identification workflow, biochemical testing, and conventional serotyping was performed; whereas in the MLMA workflow, DNA extraction, and MLMA assay followed. Procedures were performed as per protocols described above. The agreement between the MLMA assay and the conventional serological method was determined by calculating the kappa value.

### Ethics

Stool samples from which suspected isolates originated for use in this study were collected as part of laboratory-based surveillance of infectious diarrheal disease in Shenzhen, Guangdong province, China in accordance to the public health mandate, therefore ethical clearance was not required. All suspected isolates derived from stool samples were de-identified and anonymous to protect patient privacy and confidentiality.

## Results

### Detection Scheme of *V. parahaemolyticus* O and K Serogroups

A two-tube system was developed with three fluorescence channels (ROX, FAM, and Cy5) in the first tube and two fluorescence channels (ROX and FAM) in the second tube. In the first tube that detects O-serogroup antigens, the ROX channel detects antigen genes for serogroups O1, O2, O3, O4, O5, O10-*orf9*, and O11; the FAM channel detects antigen genes for serogroups O6, O7, O8, O9, O10-*wvcP*, and O12; whereas the Cy5 channel detects the *toxR* gene. In the second tube that detects K-serogroup antigens, the ROX channel detects antigen genes for serogroups K6, K8, K25, K29, K56, K68, and the *SUC2* gene as an internal control; whereas the FAM channel detects antigen genes for serogroups K3, K9, and K36. In this study, we have identified an additional gene locus, *orf9*, to be specific for serogroup O10 (Accession no: CNA0002415) and was included in the assay as a target in addition to *wvcP* for serogroup O10 identification. Table 2 details the experimental setup in each tube for the identification of all serogroups in the assay, including their gene loci, fluorophore channels, designated melting temperatures and probe sequences.

### Performance Characteristics of the MLMA Assay

**Figure 1:**
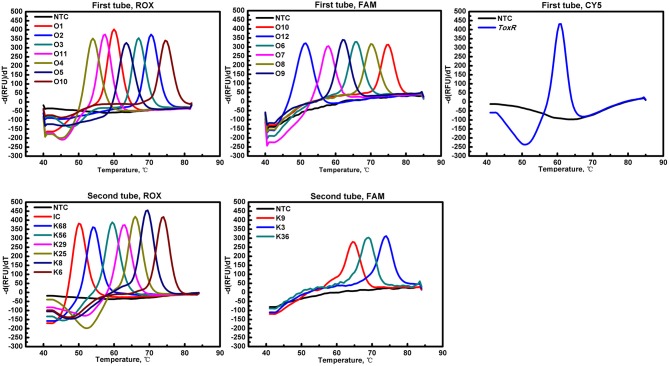
Probe melting curve analysis for the identification of *V. parahaemolyticus* O and K serogroups in the MLMA assay. Color-coded melting curves represent the different antigen genes in each of the fluorophore channels (ROX, FAM, and Cy5) in a two-tube system. IC, *SUC2* gene was used as a positive internal control (IC); NTC, negative control.

All targeted gene loci was detected by the MLMA assay, which yielded expected Tm values for each serogroup without any cross reaction (Figure 1). The limit of identification for all gene targets ranged from 0.1 to 1.0 ng/μl of DNA template for each serogroup. The standard deviation (SD) and coefficient of variation (CV) values for intra-assay and inter-assay were derived. The largest SD value for mean Tm was no more than 1°C and the CV values of the intra-assay and inter-assay ranged from 0 to 1% (Table 3).

**Table 3:** Reproducibility of designated melting temperatures (Tm) and Limit of Identification values for each serogroup target loci in the assay.

**Name**	**Gene**	**Concentration**	**Intra-assay reproducibility**			**Inter-assay reproducibility**			**Limit of identification**
		**ng/uL**	**Mean Tm (^**°**^C)**	**SD**	**CV (%)**	**Mean Tm (^**°**^C)**	**SD**	**CV (%)**	**(ng/uL)**
O1	*wvaG*	100.0	59.3	0.29	0.49	59.2	0.26	0.44	1.0
		10.0	59.3	0.29	0.49	59.3	0.27	0.45	
O2	*wvaR*	100.0	70.0	0.00	0.00	69.7	0.26	0.37	1.0
		10.0	70.0	0.00	0.00	69.8	0.27	0.38	
O3	*VP0208*	100.0	66.3	0.29	0.44	66.0	0.27	0.40	1.0
		10.0	66.2	0.29	0.44	65.9	0.35	0.54	
O4	*orf16*	100.0	54.3	0.29	0.53	54.1	0.23	0.43	1.0
		10.0	54.2	0.29	0.53	54.1	0.23	0.43	
O5	*wvcA*	100.0	63.5	0.00	0.00	63.3	0.26	0.41	1.0
		10.0	63.5	0.00	0.00	63.3	0.26	0.41	
O6	*wvcJ*	100.0	65.5	0.00	0.00	65.5	0.27	0.41	0.1
		10.0	65.8	0.58	0.88	65.6	0.42	0.64	
O7	*wvcN*	100.0	56.5	0.00	0.00	57.0	0.38	0.66	1.0
		10.0	56.5	0.00	0.00	56.8	0.26	0.46	
O8	*wvdG*	100.0	69.8	0.29	0.41	69.8	0.27	0.38	0.1
		10.0	70.0	0.00	0.00	69.8	0.38	0.54	
O9	*wvaH*	100.0	61.2	0.29	0.47	61.4	0.42	0.68	1.0
		10.0	61.2	0.29	0.47	61.4	0.35	0.58	
O10	*wvcP*	100.0	74.5	0.00	0.00	74.5	0.27	0.36	1.0
		10.0	74.5	0.00	0.00	74.5	0.00	0.00	
O10	*orf9*	100.0	74.8	0.29	0.39	74.9	0.23	0.31	1.0
		10.0	74.8	0.29	0.39	74.8	0.26	0.35	
O11	*wvdB*	100	56.3	0.29	0.51	56.6	0.32	0.57	1.0
		10.0	56.7	0.29	0.51	56.7	0.37	0.66	
O12	*wvcP*	100.0	50.5	0.00	0.00	50.7	0.37	0.73	0.1
		10.0	50.3	0.29	0.57	50.6	0.32	0.63	
IC	*SUC2*	100.0	50.7	0.29	0.57	50.6	0.17	0.33	0.1
		10.0	50.5	0.00	0.00	50.6	0.23	0.46	
VP	*toxR*	100.0	61.5	0.00	0.00	61.5	0.00	0.00	0.1
		10.0	61.5	0.00	0.00	61.6	0.27	0.27	
K3	*VP24500037*	100.0	75.10	0.28	0.38	75.10	0.22	0.29	1.0
		10.0	75.30	0.29	0.38	75.20	0.26	0.35	
K6	*VP0223*	100.0	74.30	0.29	0.39	74.40	0.22	0.30	1.0
		10.0	74.10	0.29	0.39	74.50	0.35	0.47	
K8	*VPBB0234*	100.0	70.00	0.00	0.00	70.10	0.22	0.31	1.0
		10.0	70.00	0.00	0.00	70.10	0.30	0.43	
K9	*VP13500017*	100.0	66.20	0.29	0.44	66.10	0.22	0.33	1.0
		10.0	66.50	0.00	0.00	66.30	0.25	0.38	
K25	*VP13200012*	100.0	66.80	0.29	0.43	66.90	0.17	0.25	1.0
		10.0	66.50	0.00	0.00	66.60	0.17	0.25	
K29	*VP24700016*	100.0	63.70	0.29	0.45	63.60	0.17	0.26	1.0
		10.0	63.70	0.29	0.45	63.60	0.17	0.26	
K36	*VP10400014*	100.0	70.80	0.29	0.41	70.70	0.26	0.37	1.0
		10.0	70.80	0.29	0.41	70.80	0.26	0.37	
K56	*VP33400015*	100.0	60.50	0.00	0.00	60.40	0.22	0.37	1.0
		10.0	60.30	0.29	0.48	60.30	0.25	0.41	
K68	*VP16100014*	100.0	54.30	0.29	0.53	54.40	0.17	0.31	1.0
		10.0	54.30	0.29	0.53	54.40	0.17	0.31	

### Multicenter Double-Blind Clinical Study

From a total of 6,118 consecutive diarrheal stool specimens in 2018, 153 *V. parahaemolyticus* isolates (2.5%) were identified by both traditional and MLMA workflows. Among these, 149 (97.4%) isolates belonged to 9 of the 11 serotypes identifiable by the MLMA workflow, whereas 4 (2.6%) isolates belonged to 3 other serotypes. The MLMA results demonstrated kappa values of 1.0 for each of the identifiable serotypes when compared against conventional serotyping results, indicating total agreement. A full comparison of serotype identification by MLMA and conventional serotyping is available (Table 4). In the clinical setting, the MLMA assay workflow was 12–18 h faster compared to traditional workflow.

**Table 4:** Serotype identification by MLMA assay and conventional serotyping among clinical *V. parahaemolyticus* isolates (*n* = 149) from the multicenter double-blind study.

**No**.	**Serotypes**	**Number of isolates**		**Kappa value**
		**MLMA**	**Conventional** ** serotyping**	
1	O3:K6	111	111	1
2	O4:KUT	18	18	1
3	O3:KUT	6	6	1
4	O4:K8	6	6	1
5	O1:KUT	3	3	1
6	O10:KUT	2	2	1
7	O1:K56	1	1	1
8	O4:K9	1	1	1
9	O3:K29	1	1	1

## Discussion

The transmission dynamics of *V. parahaemolyticus* infections is complex. To date, the possible mechanisms behind the emergence of a virulent pandemic serotype O3:K6 clone which has disseminated rapidly worldwide since 1996 has remained elusive (Nair et al., 2007); whilst a prominent yet unexplained upward trend in disease incidence and outbreaks of vibriosis have also been observed globally in recent years (Newton et al., 2012; Marder et al., 2017; Liu et al., 2018). Since early 1970s, the tracking of disease spread has relied on conventional serological typing, which is considered the international gold standard pertaining to *V. parahaemolyticus* epidemiology. In light of the growing importance of this foodborne pathogen and the lack of rapid and reliable molecular serotyping methods, we have developed a novel MLMA assay for the simultaneous identification of 11 clinically common *V. parahaemolyticus* serotypes.

The current assay was able to detect all gene targets (Table 3) and gave expected Tm values with robust performance characteristics. The assay has shown high reproducibility where the largest SD value for mean Tm was no more than 1°C and the CVs were no more than 1% in both intra-assay and inter-assay reproducibility analyses; whereas the detection limit ranged from 0.1 to 1 ng/μl. Overall, these performance attributes are comparable to those used for the rapid identification of several other foodborne pathogens (Jiang et al., 2017). In the multicenter double-blind clinical study we conducted, the MLMA assay has shown perfect concordance with the conventional serological method for 97.4% of all clinical isolates over a year at 10 sentinel hospitals, as indicated by kappa values of 1.0 for each of the *V. parahaemolyticus* serotypes identified. Such superior performances of the assay can be attributed to the robust MLMA methodology, which relies on the high fidelity of DNA ligase to identify gaps in nucleotide sequences of the target loci for oligonucleotide probes to fully hybridize with the flanking nucleotides before ligation could occur. We have demonstrated that the current MLMA workflow could be completed 12–18 h faster compared to the traditional workflow that relies on biochemical tests and overnight incubation for identification. From DNA extraction, both confirmation of *V. parahaemolyticus* and serotyping results can be obtained in a total assay time of 3.5 h with minimal hands-on operation. Another advantage of the assay is the applicable use of a simple boiled lysates protocol for the preparation of DNA templates, as demonstrated during the clinical study on all isolates from stool specimens. This eliminates the need for laborious procedures and the high costs associated with commercial DNA extraction and purification kits. Therefore, the current assay presents a simple and pragmatic scheme for the rapid and accurate identification of *V. parahaemolyticus* serotypes, which is especially suited for real-time applications such as outbreak situations involving large number of samples be processed quickly in a short period of time. In developing this assay, we chose 11 *V. parahaemolyticus* serotypes that represented 90% of all serotypes isolated from human infections over a 10-year period (2007–2017) in Shenzhen, China; many of which are also commonly reported among infections domestically (Han et al., 2017) and worldwide (Velazquez-Roman et al., 2014; Han et al., 2016). Given its clinical relevance, the current assay could serve as an invaluable tool for clinical and public health laboratories.

For many major foodborne pathogens, such as *Salmonella*, a plethora of molecular serotyping methods have been developed, including the use of conventional multiplex PCRs, quantitative PCRs, bead-based suspension arrays, CRISPR-based assay and pyrosequencing assay (Munoz et al., 2010; Li R. et al., 2014; Aydin et al., 2018; Bugarel et al., 2018; Furukawa et al., 2018; Heymans et al., 2018). However, the lack of molecular serotyping described for *V. parahaemolyticus* has been remarkable, given the growing public health importance of this pathogen. To our knowledge, only four studies have explored the use of molecular methods for serogroup or serotype identification of *V. parahaemolyticus* to-date. Conventional PCR-based assays have been developed for the identification of O-serogroups (Chen et al., 2012; Guo et al., 2017) and serotype O3:K6 (Yeung et al., 2003), whereas the utility of MALDI-ToF has been explored for the identification for serotype O4:K8 (Li et al., 2018). However, the practical application of these methods has significant limitations, such as the need for cumbersome procedures associated with gel electrophoresis and the ability to only identify limited or single serogroups and serotypes simultaneously. Taken together, the lack of developments hitherto and the disadvantages of existing methods have prompted the urgent need for a pragmatic molecular serotyping method for *V. parahaemolyticus*.

During the development of the current assay, we chose genes encoding for the somatic (O) antigen and the capsular (K) antigen as our target genes for identification of *V. parahaemolyticus* O-serogroups (Chen et al., 2012) and K-serogroups (Chen et al., 2010), respectively. As such, the allelic heterogeneity indexed would closely mirror that of the conventional serological method. This has the advantage over the use of arbitrary molecular markers from other gene loci for serotype prediction or correlation. It is noteworthy that the lipopolysaccharide (LPS) in the outer membrane of *V. parahaemolyticus* does not possess the O-side chain (Hashii et al., 2003) that consists of oligosaccharide repeats that forms the basis of antigenic variation of the O antigen in many other Gram negative bacteria. Instead, the serological O-specificity and the determinant of *V. parahaemolyticus* O-serogroups is attributable to the core oligosaccharide of the LPS (Iguchi et al., 1995), referred to as the OGD (Chen et al., 2012). In reviewing the OGD for all serogroups during the study, we have discovered that in addition to the *wvcP* gene, the *orf9* locus was also a genetic determinant for serogroup O10. Subsequently, *orf9* was incorporated into our assay to complement the use of *wvcP* to provide a robust support for the identification of serogroup O10. Indeed, a better understanding of the OGD through genetic analyses would further improve the current O-antigenic scheme. A recent study has shown that the frequent occurrence of untypeable O-serogroups (OUTs) may have resulted from intra-species recombination events that occurred within the OGD region and evolved from the current O-serogroups, where several provisional and rare O-serogroup candidates were proposed (Guo et al., 2017). Whereas, for K- serogroups, the genetic locations for encoding K-antigen synthesis in *V. parahaemolyticus* (Guvener and Mccarter, 2003; Okura et al., 2003) have been a subject of debate, but was subsequently confirmed to be between *gmhD* and *rjg* using an O3:K6 isolate by construction of gene deletions (Chen et al., 2010). By using the genetic location as reference during sequence analyses for assay development, our assay was able to produce robust identification for additional K-serogroups (K8, K25, K29, K56, K68) and further corroborated these findings. Indeed, our current study presented the first molecular assay for the identification of *V. parahaemolyticus* K-serogroups on the basis of the K- antigen synthesis gene locus; as well as being used in conjunction of O- serogroup identification for the first time to provide the molecular identification of *V. parahaemolyticus* O:K serotypes.

There are a number of aspects in which the current assay could be further improved. The rare O13 serogroup was not included for identification in the current assay, owing to the 100% homology of the corresponding regions with the O3 serogroup (Makino et al., 2003), as was found in a conventional PCR method that could not differentiate O3 and O13 serogroups (Chen et al., 2012). Further work would be needed to identify heterogeneous genetic markers for the accurate identification of the O13 serogroup. Additionally, the inclusion of additional K- serogroups in response to the rapidly evolving nature of K antigens would further increase the utility of the assay. Finally, a useful aspect worth exploring would be the direct application of clinical and food samples by incorporating inhibitor resistant polymerases during the amplification of target genes, further reducing processing time from the assay workflow.

## Conclusion

This is the first description of a molecular assay for the simultaneous identification of multiple clinically common *V. parahaemolyticus* serotypes. The current assay satisfies the urgent need for a practical, rapid, and robust identification of *V. parahaemolyticus* serotypes. Given the marked rise in the incidence of vibriosis in recent years, it is anticipated that the current assay could contribute significantly for the timely and effective public health intervention of vibriosis.

## Data Availability Statement

All datasets generated for this study are included in the article/Supplementary Material.

## Author Contributions

ML and YJ conceived and designed the experiment. ML, YLin, YQ, LZ, YD, and ZL performed the experiments. ML, XS, YLi, MJ, YLiao, and QL contributed analysis. ML and QH wrote the paper.

### Conflict of Interest

The authors declare that the research was conducted in the absence of any commercial or financial relationships that could be construed as a potential conflict of interest.

## Abbreviations

MLMA: Multiplex Ligation Reaction based on Probe Melting Curve Analysis
FAM: carboxy fluorescein
ROX: carboxy-X-rhodamine
Cy5: indodicarbocyanine5
LATE PCR: Linear After-The-Exponential PCR
Tm: melting temperature
IC: internal control.
