# Supplementary material for: Simultaneous Identification of Clinically Common Vibrio parahaemolyticus Serotypes Using Probe Melting Curve Analysis
Source: Front Cell Infect Microbiol. 2019 Nov 14;9:385. doi: 10.3389/fcimb.2019.00385 (PMC6868019; doi:10.3389/fcimb.2019.00385)
Supplement: Supplementary file 1 [file Table_1.pdf]

SUPPLEMENTAL TABLE

Table S1. Additional 38 *V. parahaemolyticus* serotypes (n=66) used for detecting cross reactions

Serotype	Number of isolates	Source
O1:K1	1	Shenzhen CDC
O4:K4	1	Shenzhen CDC
O3:K5	1	Shenzhen CDC
O4:K11	1	Shenzhen CDC
O4:K12	1	Shenzhen CDC
O4:K13	1	Shenzhen CDC
O11:K19	1	Shenzhen CDC
O11:K20	1	Shenzhen CDC
O1:K23	1	Shenzhen CDC
O2:K28	1	Shenzhen CDC
O5:K30	1	Shenzhen CDC
O3:K31	1	Shenzhen CDC
O1:K32	1	Shenzhen CDC
O1:K33	1	Shenzhen CDC
O4:K34	1	Shenzhen CDC
O3:K37	1	Shenzhen CDC
O1:K38	1	Shenzhen CDC
O4:K42	1	Shenzhen CDC
O3:K48	1	Shenzhen CDC
O4:K49	1	Shenzhen CDC
O4:K55	1	Shenzhen CDC
O4:K63	1	Shenzhen CDC
O10:K66	1	Shenzhen CDC
O1:K69	1	Shenzhen CDC
O10:K71	1	Shenzhen CDC
O6:KUT	1	Shenzhen CDC
O8:KUT	1	Shenzhen CDC
O5:KUT	1	Shenzhen CDC
O10:KUT	2	Shenzhen CDC
O2:KUT	2	Shenzhen CDC
O8:K21	2	Shenzhen CDC
O4:KUT	3	Shenzhen CDC
O8:K41	3	Shenzhen CDC
O10:K60	3	Shenzhen CDC
O11:KUT	3	Shenzhen CDC
O6:K18	4	Shenzhen CDC

O5:K17	8	Shenzhen CDC
O9:K44	8	Shenzhen CDC
O7[a]	1	*Escherichia coli* TOP10 strain
O12[a]	1	*Escherichia coli* TOP10 strain

[a] O-serogroup antigen genes, O7-*wvcN* and O12-*wvcP*, were each cloned into a pUC57 vector and maintained in a *Escherichia coli* TOP10 strain to represent *V. parahaemolyticus* O7 and O12 serogroups respectively.
